# The association between change of *soluble tumor necrosis factor receptor R1 (sTNF-R1)* measurements and cardiovascular and all-cause mortality—Results from the population-based (Cardiovascular Disease, Living and Ageing in Halle) CARLA study 2002–2016

**DOI:** 10.1371/journal.pone.0241213

**Published:** 2020-10-26

**Authors:** Lamiaa Hassan, Daniel Medenwald, Daniel Tiller, Alexander Kluttig, Beatrice Ludwig-Kraus, Frank Bernhard Kraus, Karin H. Greiser, Rafael Mikolajczyk

**Affiliations:** 1 Institute of Medical Epidemiology, Biometrics and Informatics, Interdisciplinary Center for Health Sciences, Martin-Luther-University Halle-Wittenberg, Halle/Saale, Saxony-Anhalt, Germany; 2 Department of Radiation Oncology, University Hospital Halle, Halle/Saale, Saxony-Anhalt, Germany; 3 Central Laboratory, University Hospital Halle, Halle/Saale, Saxony-Anhalt, Germany; 4 German Cancer Research Centre, Division of Cancer Epidemiology, Heidelberg, Baden-Württemberg, Germany; Kaohsiung Medical University Hospital, TAIWAN

## Abstract

**Aims:**

Single measurements of higher levels of soluble tumor necrosis factor receptor I (sTNF-R1) have been shown to be associated with increased risk of mortality. However, up to date, little is known about the underlying temporal dynamics of sTNF-R1 concentrations and their relation with mortality. We aimed to characterize the effect of changes in sTNFR-1 levels on all-cause and cardiovascular mortality, independent from other established risk factors for mortality, including other inflammatory markers.

**Methods:**

We used data of the population based cohort study CARLA and included 1408 subjects with sTNF-R1 measured at baseline (2002–2006) and first follow-up (2007–2010). Cox proportional hazard models were used to assess the association of baseline and follow-up sTNF-R1 measurements with all-cause and cardiovascular mortality during ~10 years since the first follow-up after adjusting for relevant confounders.

**Results:**

Based on 211 deaths among 1408 subjects, per each doubling of the baseline sTNF-R1, the risk of all-cause mortality was increased by about 30% (Hazard ratio 1.28, 95% Confidence Interval 0.6–2.7), while per each doubling of the follow-up level of sTNF-R1 mortality was 3-fold (3.11, 1.5–6.5) higher in a model including both measurements and adjusting for confounders. The results were mainly related to the cardiovascular mortality (5.9, 2.1–16.8 per each doubling of follow up sTNF-R1 value).

**Conclusion:**

Solely the follow-up value, rather than its change from baseline, predicted future mortality. Thus, while sTNF-R1 levels are associated with mortality, particularly cardiovascular, over a long-time period in the general population, if they change, the earlier measurements play no or little role.

## Introduction

Human ageing is characterized by a chronic, sterile (in the absence of an infection), low-grade inflammation, and this phenomenon has been termed "inflammaging" [1]. A possible mechanism of inflammaging is the continuous stimulation of macrophages by molecular debris whose generation–discarding equilibrium becomes compromised with ageing [2]. Macrophages are the major source of tumor necrosis factor alpha (TNFα) [3], which is a cytokine with a wide range of pro-inflammatory bioactivities. It exerts its function via binding to and activation of TNF receptor 1 (TNF-R1) among others [4]. One model of TNF-R1 signaling suggests that TNF-R1 predominantly promotes inflammation and tissue degeneration [5]. Soluble tumor necrosis factor receptor 1 (sTNF-R1) is the circulating form of their membrane bound counterparts [6]. Several studies demonstrated increased all-cause and cardiovascular mortality in patients with various diseases and higher levels of sTNF-R1 [7–12]. In the general population, elevated levels of sTNF-R1 were associated with all-cause and cardiovascular mortality after adjustment for lifestyle factors, inflammation due to chronic diseases, and established cardiovascular risk factors [13–15].

For three other inflammatory biomarkers, C-reactive protein (CRP), interleukin-6 (IL-6), and α1-acid glycoprotein (AGP), it was shown that the association with mortality is weakened over time since measurement [16–19]. Also, for CRP, it was shown that if a more recent measurement is available, then the earlier measurement does not provide additional information [20, 21], which suggests that the marker might not be associated with an irreversible damage, at least not in individuals who experience a change in their CRP levels. However, sTNF-R1 might differ from CRP, which is also driven by acute inflammation.

While several studies assessed sTNF-R1 as a biomarker of ageing [14, 22, 23], most of them studied populations with specific diseases and were limited to a single measurement of sTNF-R1. In this study, we aim to assess the association of sTNF-R1 with all-cause and cardiovascular mortality in general population and to examine what are the effects of changes in sTNF-R1 levels occurring during the follow-up time.

## Material and methods

### Study population

**Fig 1:**
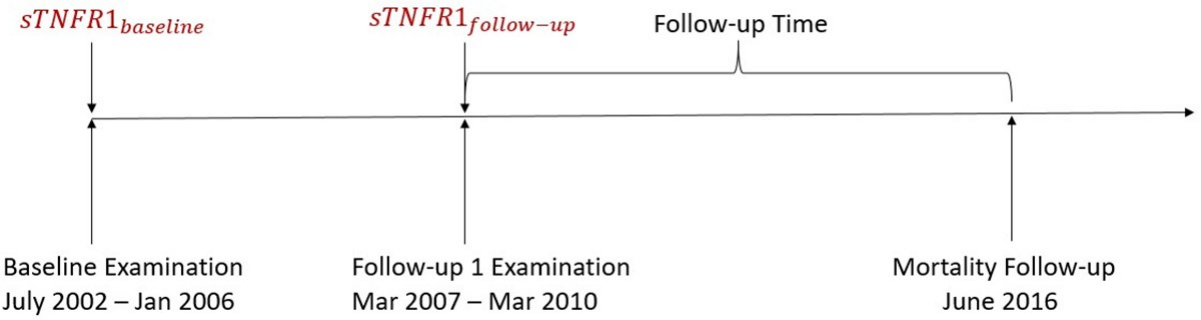
Timeline of the CARLA study.

We used data from the CARdiovascular Living and Ageing in Halle study (CARLA study), which is a prospective cohort study in the general population of the city of Halle, Eastern Germany. Details of the CARLA study have been described previously [24, 25]. In short, the CARLA cohort includes 1,779 subjects (812 women and 967 men) aged 45–83 years at baseline in 2002. They were recruited from general population using registration data. For the present study, we included all subjects who took part in the baseline and first follow-up examination (N = 1408) and had complete data on sTNF-R1 both at baseline and at the first follow-up examination (Fig 1). The original study was approved by the Ethics Committee of the Medical Faculty of the Martin-Luther University Halle-Wittenberg and by the State Data Privacy Commissioner of Saxony-Anhalt and conforms to the principles outlined in the Declaration of Helsinki. All subjects gave written informed consent.

### Mortality follow-up

The primary outcome was all-cause mortality, while the secondary outcome was cardiovascular mortality. For all subjects included in our study, mortality was recorded from the date of the first follow-up examination (2007–2010) until June 2016 (Fig 1). The cause of death was defined as specified in the official death certificate compiled by the Federal Statistical Office. The cause of death was initially recorded by a medical doctor and subsequently reviewed by a certified coder at the State Statistical Office of Saxony-Anhalt (Statistisches Landesamt Sachsen Anhalt). Cardiovascular mortality was defined as ICD-10: I10-I79.

### Covariates

**Fig 2:**
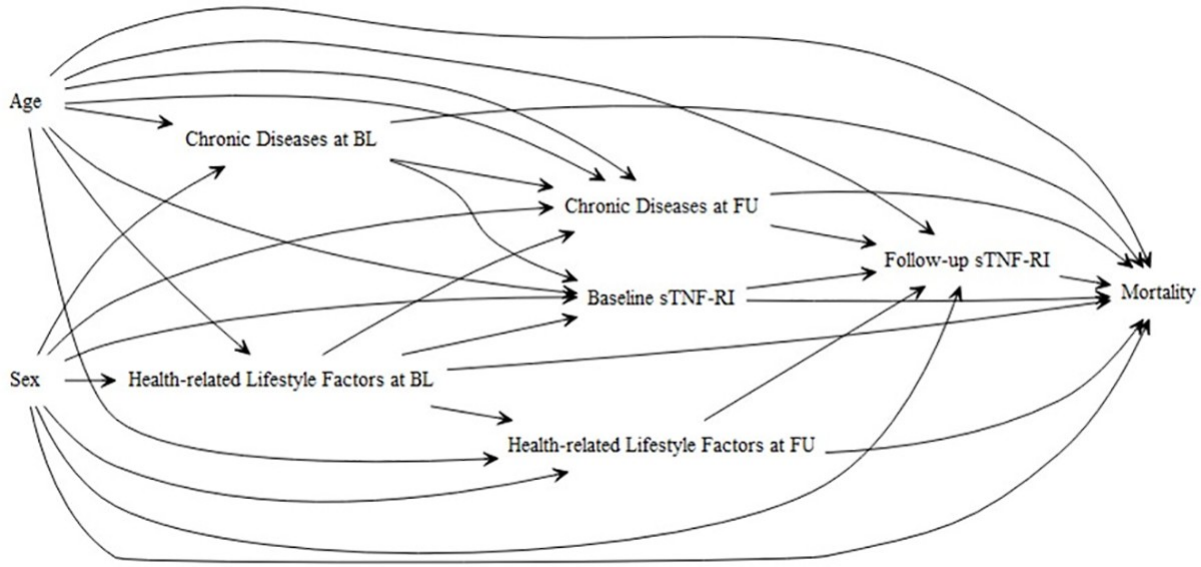
Directed acrylic graph (DAG) used for the analysis.

We assessed the effects of sTNF-R1 measured at baseline and at follow-up on the mortality after follow up. We selected covariates based on a directed acyclic graph (DAG) for parameters known to be associated with inflammation as well as mortality (Fig 2, Table 1). As our focus was on inflammation beyond the effects of known diseases of the patient affecting mortality and inflammation, we adjusted only for the reported diseases. We used the covariate information from the first follow-up examination, but also conducted sensitivity analysis in a sample restricted to subjects in whom no change in covariates, which were included in the DAG, occurred between baseline and follow-up.

**Table 1:** Characteristics of 1,408 CARLA subjects at the first follow-up examination.

Variables		Alive (N = 1197)	Deceased (N = 211)
Age (years), Mean (SD)		65.8 (9)	75.5 (9)
Sex (Male)		625 (52.2%)	155 (73.5%)
BMI (kg/m^2^), Mean (SD)		28.3 (5)	28.8 (5)
sTNF-R1 (pg/mL), Median (IQR)		1175.9 (362)	1514.0 (749)
IL-6 (pg/mL), Median (IQR)		2.3 (3)	3.6 (3)
hsCRP (mg/L), Median (IQR)		1.7 (2)	2.2 (4)
eGFR (mL/min/1.73 m2), Mean (SD)		86.3 (21)	75.6 (27)
Low-density lipoprotein (LDL) (mmol/l), Mean (SD)		3.4 (1)	3.2 (1)
High-density lipoprotein (HDL) (mmol/l), Mean (SD)		1.4 (0)	1.3 (0)
Total cholesterol (mmol/l), Mean (SD)		5.5 (1)	5.3 (1)
Triglycerides (mmol/l), Mean (SD)		1.9 (1)	2.0 (1)
Sport Index, Mean (SD)		2.5 (0.8)	2.3 (0.7)
**Prevalence of**			
Chronic Heart Failure		92 (7.7%)	53 (2.5%)
Myocardial Infarction		67 (5.6%)	32 (15.2%)
Stroke		40 (3.3%)	26 (12.3%)
Hypertension		911 (76.1%)	193 (91.5%)
Diabetes Mellitus Type 2		186 (15.5%)	64 (30.3%)
Rheumatism		113 (9.4%)	22 (10.4%)
Cancer		106 (8.9%)	44 (20.9%)
Thyroid Disease		315 (26.3%)	58 (27.5%)
Thyroid Medication		227 (19%)	37 (17.5%)
Lipids/ Statins Medication		272 (22.7%)	67 (31.8%)
Antihypertensive Medication		731 (61.1%)	174 (82.5%)
Antiplatelet Medication		232 (19.4%)	72 (34.1%)
Smoking	Never	545 (45.5%)	62 (29.4%)
	Current	166 (13.9%)	23 (10.9%)
	Occasional	27 (2.3%)	4 (1.9%)
	Ex-Smoker	455 (38.0%)	119 (56.4%)

For the covariates, we used the following definitions: Chronic heart failure was defined in accordance with the algorithm introduced by the European Society of Cardiology [26], taking clinical symptoms, the plasma levels of NT-pro brain natriuretic peptide (NT-proBNP), and echocardiographic findings into account [27]. Blood pressure was assessed by taking three consecutive measurements after a resting period of at least 5 min. Weight and height were measured using the SECA-107 digital scale and SECA-220 height measuring system [24], respectively. The estimated glomerular filtration rate (eGFR) was assessed using the formula by the Chronic Kidney Disease Epidemiology Collaboration (CKD-EPI formula) [28].

Self-reported information about physician-diagnosed diabetes, rheumatic diseases, cancer, cardiovascular diseases (coronary heart disease, stroke, and arterial hypertension) and thyroid disorders (hyper- and hypothyroidism, thyroid nodules, goiter and Graves’ Disease) was collected through a computer-based interview. Information on the use of medication during the seven days preceding the examination was collected by the study nurse using the computer-based IDOM program and was used for definition of diabetes and hypertension [29].

### Laboratory measurements

The Central Laboratory at the University Hospital of Halle (Saale) undertook the determination of sTNF-R1 levels. The analyzed specimen were serum samples, stored at -80°C before measurement. Measurement of sTNF-R1 concentrations was carried out using the Human sTNF-R1/TNFSF1A Immunoassay Quantikine® ELISA (R&D Systems) on an Epoch 2 Microplate Spectrophotometer (BioTek, Bad Friedrichshall, Germany). sTNF-R1 concentrations were measured in double with standard quality controls (Quantikine Immunoassay Control Group; R&D Systems) and calibrations for each used microplate in accordance with the recommendations of the manufacturer. Interleukin-6 (IL-6) measurements were carried out on a Roche cobas e602 modular analyzer (Roche Diagnostics, Mannheim, Germany) using the IL-6 electrochemiluminescence immunoassay (ECLIA) kit from Roche (Elecsys IL-6, Roche, Mannheim, Germany). The cobas e602 analyzer and the used reagents were operated and calibrated according to the manufacturer’s instructions and manuals, with routine maintenance and quality control procedures. The determination of CRP was undertaken by the Institute of Laboratory Medicine, Clinical Chemistry and Molecular Diagnostics at the Leipzig University Clinics. The laboratory has been accredited according to the accreditation norms ISO 15180 and ISO 17025. Serum levels of high-sensitivity CRP (hsCRP) were measured using a high-sensitivity immunoturbidimetric method (CRP [Latex] HS, Roche, Mannheim, Germany) on a Hitachi autoanalyzer (Roche Diagnostics, Mannheim, Germany). Laboratory analyses of non-fasting venous blood samples included serum total, high-density (HDL) and low-density (LDL) lipoprotein cholesterol, triglycerides from EDTA-blood.

### Statistical analysis

General descriptive statistics were calculated for the baseline characteristics of the population. Continuous variables were displayed as calculated means with their standard deviation or as medians with interquartile range (IQR) in the case of skewed distributions. Categorical variables were displayed as numbers and percentages.

To accommodate skewness of the baseline and follow-up measurements of sTNF-R1, both were modeled as continuous variables using a log (base 2) transformation, which corresponds to one unit expressing the doubling of sTNF-R1 value.

Cox proportional hazard regression was used to estimate the hazard ratios (HR) and 95% confidence intervals (CI) of all-cause and cardiovascular mortality resulting from inflammaging. The assumption of proportionality of hazards was confirmed by assessing the Schoenfeld residuals, which showed no non-zero slopes (S1 Fig). We also tested the form of the association between both sTNF-R1 values and the hazard of mortality using generalized additive model (GAM) (S2 and S3 Figs).

In order to assess the effects of changes in sTNF-R1 on subsequent mortality, we first studied the sTNF-R1 measurements at baseline and at follow-up and their interaction in a joint model. This specification allows differentiating between the effects of both measurements and provides insights for whether change should be considered. Model 1 included no further covariates; in further analyses, we included covariates in a stepwise manner. Covariates were first grouped in two separate groups to differentiate the underlying effects of the health-related lifestyle risk factors versus the effects of the chronic diseases and their indicators (Model 2 vs. Model 3). Both groups of covariates were included together in Model 4. To assess specific effects of sTNF-R1, we additionally included in Model 5 two other main inflammation parameters, hsCRP and Interleukin-6 (IL-6). In order to differentiate between acute and chronic inflammation, we conducted a sensitivity analysis in which subjects with hsCRP plasma level >10 mg/L at baseline and/or follow-up were excluded. Furthermore, we conducted another sensitivity analysis where we included only subjects who had stable levels for all covariates listed in Table 1 except age and sex, sTNF-R1 and thyroid disease. For the continuous variables, we included subjects who had less than ±10% change relative to their baseline measurement. Cause-specific hazard ratios were estimated for the cardiovascular mortality risk to accommodate for the presence of competing risks using the r package “riskRegression” [30].

All statistical analyses were conducted using R, version 3.5.0 [31].

## Results

### Baseline clinical characteristics

Out of 1408 subjects, 211 died (including 89 (= 42%) cardiovascular deaths) during a mean duration of follow-up time of 7.4 years from the first follow-up examination (Table 1). Among the deceased, the majority (n = 155) were men with a mean age at time of death of 74.8 years; in comparison, female subjects (n = 56) died at the mean age of 77.2 years. Among those who died, nearly all investigated clinical and subclinical conditions, including higher levels of all three inflammatory markers sTNF-R1, hsCRP and IL-6, were more common than among those who survived (Table 1). Prevalence of thyroid disorders was relatively similar in both groups shown in Table 1. Among those who were still alive at the end of follow-up time 227 took thyroid medication, 73% took sodium levothyroxine and 15% took levothyroxine and potassium iodide, 6% took only potassium iodide. While among those who died, 37 took thyroid medication, 70% took sodium levothyroxine, 11% took levothyroxine, and potassium iodide and 11% took only potassium iodide.

### Distribution of sTNF-R1 levels at baseline and follow-up

Mean sTNF-R1 increased on average from baseline to follow-up. The mean of the absolute change was 185.18 pg/ml (with a standard deviation of 297.9 pg/ml), which corresponds to 16.3% of the mean baseline value. Among all patients, 89% stayed in the range of +/-5% of their baseline value (on the log2 scale) while 11% had more than 5% increase and 0.3% experienced more than 5% decrease of their baseline value.

### Effects of baseline and follow-up sTNF-R1 levels on overall and cardiovascular mortality

There was no indication of interaction between baseline and follow-up values (data not shown), interaction effect was therefore not considered in further analyses. When follow-up sTNF-R1 value was included in the model, baseline sTNF-R1 played little role (Table 2). In model 5, for each doubling of the baseline sTNF-R1 the risk increased by about 30% (HR = 1.28, 95% CI = (0.6–2.8)), while for each doubling of the follow-up level of sTNF-R1, mortality risk increased 3-fold (HR = 3.00, 95% CI = (1.4–6.3)). Thus, not the change, but solely the changed value was relevant.

**Table 2:** Association of baseline and follow-up sTNF-R1 measurements with all-cause mortality estimated by using Cox regression models.

		Adjusted HR (95% CI) Model 1	Adjusted HR (95% CI) Model 2	Adjusted HR (95% CI) Model 3	Adjusted HR (95% CI) Model 4	Adjusted HR (95% CI) Model 5 With CRP/IL-6
**All Subjects**	**Baseline sTNF-R1**	1.56 (0.9–2.8)	1.57 (0.9–2.9)	1.59 (0.9–2.9)	1.55 (0.8–2.9)	1.73 (0.9–3.5)
	**Follow-up sTNF-R1**	1.99 (1.2–3.2)	1.80 (1.1–3.0)	2.77 (1.7–4.7)	2.67 (1.5–4.8)	2.16 (1.1–4.1)
**Subjects without signs of acute inflammation** *	**Baseline sTNF-R1**	1.23 (0.6–2.4)	1.10 (0.5–2.3)	1.19 (0.6–2.4)	1.11 (0.5–2.3)	1.28 (0.6–2.8)
	**Follow-up sTNF-R1**	2.33 (1.3–4.2)	2.24 (1.2–4.1)	3.60 (1.8–7.1)	3.41 (1.7–6.9)	3.00 (1.4–6.3)

Baseline and Follow-up measurements were included in each model. Model 1 is adjusted for age, sex. Model 2 is adjusted for age, sex and lifestyle risk factors: BMI, smoking, physical activity. Model 3 is adjusted for age, sex, and comorbidities: GFR value, hypertension, myocardial infarction (self-reported), diabetes mellitus 2, chronic heart failure, cancer, rheumatism and medication intake. Model 4 is adjusted for age, sex, lifestyle risk factors and comorbidities. Model 5 is adjusted for all covariates in Model 4 and additionally adjusted for IL-6, hsCRP, triglycerides, total, low- and high-density lipoprotein cholesterol level. HR Hazard Ratio, CI Confidence Interval. Bold indicates statistical significance.

*All subjects with hsCRP values > 10 mg/L at either baseline or follow-up or both were excluded.

In the sensitivity analysis that included 786 subjects who had same covariates levels at baseline and follow-up, the estimates of the follow-up measurement maintained the same magnitude: for subjects without signs of acute inflammation each doubling of the follow-up sTNF-R1 increased mortality risk by almost 4-fold (HR = 3.99, 95% CI = (1.4–12.7) (Table 3). While the effect estimates of the follow-up sTNF-R1 in the sensitivity analysis were slightly changed, they remained within the same magnitude of the effect estimates in the main analysis of the whole study population. This indicates that adjustment for follow up covariates in the main analysis is not responsible for the stronger effects of follow-up sTNF-R1. The results were similar for the cardiovascular mortality, with a stronger association between follow-up sTNF-R1 and mortality (Table 4), while for the non-cardiovascular mortality, after adjusting for all covariates, both baseline (HR = 2.07, 95% CI = (0.7–6.0)) and follow-up estimates of sTNF-R1 (HR = 1.38, 95% CI = (0.4–3.9)) (data not shown) had confidence intervals that included one.

**Table 3:** Association of baseline and follow-up sTNF-R1 measurements with all-cause mortality for 768 subjects with stable/unchanged covariates.

		Adjusted HR (95% CI) Model 1	Adjusted HR (95% CI) Model 2	Adjusted HR (95% CI) Model 3	Adjusted HR (95% CI) Model 4	Adjusted HR (95% CI) Model 5 With CRP/IL-6
**All Subjects**	**Baseline sTNF-R1**	1.32 (0.6–2.8)	1.64 (0.7–4.1)	1.25 (0.5–3.0)	1.57 (0.6–4.0)	1.64 (0.6–4.3)
	**Follow-up sTNF-R1**	2.09 (1.0–4.5)	1.45 (0.6–3.4)	3.68 (1.6–8.5)	2.25 (0.9–5.6)	1.97 (0.8–5.2)
**Subjects without signs of acute inflammation** *	**Baseline sTNF-R1**	0.98 (0.3–2.7)	1.04 (0.4–2.9)	0.77 (0.3–2.3)	0.84 (0.3–2.6)	0.89 (0.3–2.7)
	**Follow-up sTNF-R1**	2.28 (0.9–5.8)	1.96 (0.7–5.2)	4.42 (1.5–12.9)	3.73 (1.2–11.2)	3.99 (1.4–12.7)

Baseline and Follow-up measurements were included in each model. Model 1 is adjusted for age, sex. Model 2 is adjusted for age, sex and lifestyle risk factors: BMI, smoking, physical activity. Model 3 is adjusted for age, sex, and comorbidities: GFR value, hypertension, myocardial infarction (self-reported), diabetes mellitus 2, chronic heart failure, cancer, rheumatism and medication intake. Model 4 is adjusted for age, sex, lifestyle risk factors and comorbidities. Model 5 is adjusted for all covariates in Model 4 and additionally adjusted for IL-6, hsCRP, triglycerides, total, low- and high-density lipoprotein cholesterol level. HR Hazard Ratio, CI Confidence Interval. Bold indicates statistical significance.

*All subjects with hsCRP values > 10 mg/L at either baseline or follow-up or both were exclude.

**Table 4:** Association of baseline and follow-up sTNF-R1 measurements with cardiovascular mortality estimated by using Cox regression models.

		Adjusted cause-specific HR (95% CI) Model 1	Adjusted cause-specific HR (95% CI) Model 2	Adjusted cause-specific HR (95% CI) Model 3	Adjusted cause-specific HR (95% CI) Model 4	Adjusted cause-specific H (95% CI) Model 5 With CRP/IL-6
**All Subjects**	**Baseline sTNF-R1**	1.22 (0.5–3.0)	1.15 (0.5–2.9)	1.25 (0.5–3.1)	1.26 (0.5–3.2)	1.70 (0.6–4.9)
	**Follow-up sTNF-R1**	2.76 (1.4–5.3)	2.76 (1.4–5.5)	3.76 (1.8–8.1)	4.13 (1.8–9.6)	2.93 (1.1–7.9)
**Subjects without signs of acute inflammation** *	**Baseline sTNF-R1**	0.70 (0.3–2.0)	0.60 (0.2–1.6)	0.60 (0.2–1.8)	0.60 (0.2–1.8)	0.9 (0.3–2.7)
	**Follow-up sTNF-R1**	5.33 (2.4–11.9)	4.93 (2.2–11.1)	9.5 (3.6–25.2)	8.7 (3.2–23.7)	6.5 (2.2–19.1)

Baseline and Follow-up measurements were included in each model. Model 1 is adjusted for age, sex. Model 2 is adjusted for age, sex and lifestyle risk factors: BMI, smoking, physical activity. Model 3 is adjusted for age, sex, and comorbidities: GFR value, hypertension, myocardial infarction (self-reported), diabetes mellitus 2, chronic heart failure, cancer, rheumatism and medication intake. Model 4 is adjusted for age, sex, lifestyle risk factors and comorbidities. Model 5 is adjusted for all covariates in Model 4 and additionally adjusted for IL-6, hsCRP, triglycerides, total, low- and high-density lipoprotein cholesterol level. HR Hazard Ratio, CI Confidence Interval. Bold indicates statistical significance.

*All subjects with hsCRP values > 10 mg/L at either baseline or follow-up or both were excluded.

## Discussion

In a general population cohort, with sTNF-R1 measured at baseline and four years later, we confirmed previous findings regarding association of this marker with mortality, mostly cardiovascular, but also demonstrated that if sTNF-R1 value changes, then the more recent value is more strongly associated with mortality, consistent with absence of chronic or irreversible damage.

While many studies [14, 32] assessed association with mortality for a single measurement of a biomarker of inflammaging, only few studies used multiple measurements and none of the studies analyzed sTNF-R1 [20, 21, 33–36]. For both hsCRP and IL6, previous studies indicated that only the changed value plays a role, and once the follow-up measurement is accounted for, the baseline measurement does not predict mortality [20, 21]. This would indicate a similar effect to our analysis. hsCRP is also known to display acute inflammation, but previous studies excluded subjects with high hsCRP values to account for it, as we did in our sensitivity analysis. Still, sTNF-R1 was identified as a long-time predictor of mortality in previous studies [13, 14]. The combination of long-term prediction and lack of cumulative effects (as indication of chronic damage) suggest that the long-term effects can be explained by a fraction with stable sTNF-R1 values. Once the sTNF-R1 value changes, the new level is the one determining the risk of mortality. This also means that recovery from inflammation is possible–the prediction of mortality is related to the probability that inflammaging will persist, not to the fact that it was present at one point.

We also found that sTNF-R1 is linked mainly to cardiovascular mortality, and the effects seen for overall mortality appear just the consequence of including cardiovascular mortality in overall mortality.

The prevalence of thyroid disorders observed in our cohort is in line with the prevalence observed in other studies in Germany. Schumm-Dräger et al. reported that almost a quarter of the adult German population has a nodular goiter [37]. Among a sample of elderly people, 57% showed morphological thyroid abnormalities [38].

### Study strengths and limitations

Strengths of our study include the population-based sample with more than a decade of follow-up for survival outcomes. However, several limitations deserve mention. In our study, to be able to analyze the effect of change of sTNF-R1 on mortality, we had to include solely the CARLA participants that had biomarker measurements at baseline and at first follow-up. That means that the participants who did not survive to the first follow-up examination or the participants who did not take part in the first follow-up examination could not be included. Although the response rate of the first follow-up examination was high (92%) [39], the inherent survivorship bias remains and hence the findings should be interpreted with caution. Moreover, we only had measurements of sTNF-R1 at two time points, which cannot fully capture trajectories over time. The more detailed classification of mortality was not possible due to the low number of deceased subjects. Our results apply to the general population above an age of 45 and thus are only generalizable to collectives with a similar age and cardiovascular risk profile.

## Conclusion

sTNF-R1 levels are associated with mortality over a long-time period in the general population, but if they change, only the more recent measurement is associated with mortality, indicating lack of irreversible damage. Future studies including more time points at which sTNF-R1 is measured are necessary to confirm or falsify our findings. In addition, a better understanding of how inflammaging may affect risk of mortality would help to formulate specific hypotheses.

## Supporting information

S1 Fig Schoenefeld residuals of log2 (sTNF-R1) measurements at baseline and follow-up. (TIF)

S2 Fig Spline curve of the association between the soluble receptor for tumor necrosis factor-receptor 1 (sTNF-R1) at baseline centred per (pg/mL) and mortality as hazard ratio. Each simulation is plotted using a separate light blue line.
(TIF)

S3 Fig Spline curve of the association between the soluble receptor for tumor necrosis factor-receptor 1 (sTNF-R1) at follow-up centred per (pg/mL) and mortality as hazard ratio. Each simulation is plotted using a separate light blue line.
(TIF)
