# Supplementary figures and images for: The association between change of soluble tumor necrosis factor receptor R1 (sTNF-R1) measurements and cardiovascular and all-cause mortality—Results from the population-based (Cardiovascular Disease, Living and Ageing in Halle) CARLA study 2002–2016
Source: PLoS One. 2020 Oct 26;15(10):e0241213. doi: 10.1371/journal.pone.0241213 (PMC7588092; doi:10.1371/journal.pone.0241213)

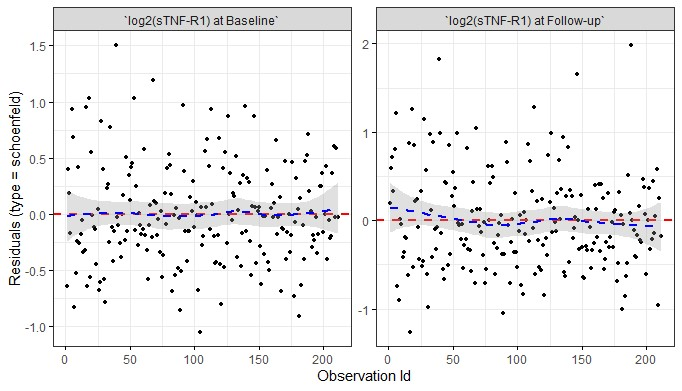

Supplement: S1 Fig — (TIF) [file pone.0241213.s001.tif]

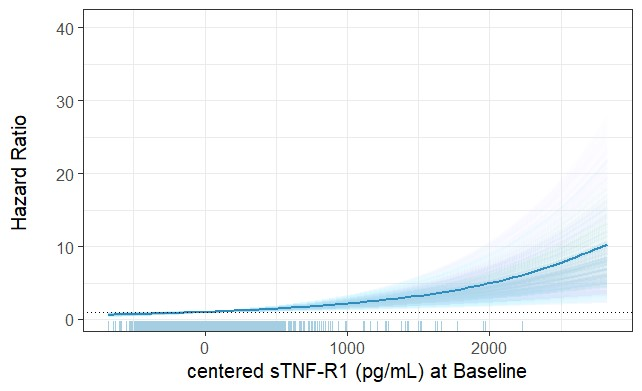

Supplement: S2 Fig — Each simulation is plotted using a separate light blue line. (TIF) [file pone.0241213.s002.tif]

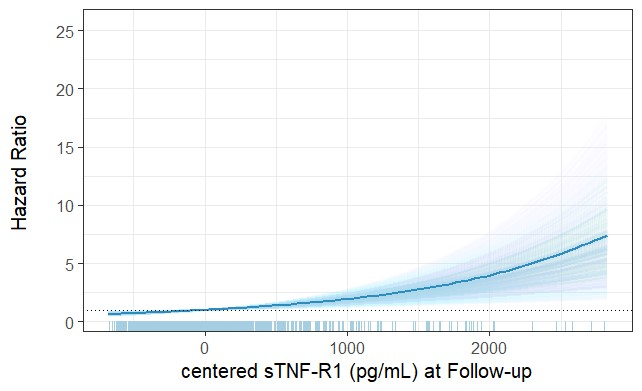

Supplement: S3 Fig — Each simulation is plotted using a separate light blue line. (TIF) [file pone.0241213.s003.tif]
